# Editorial: Neuroinflammation and Its Resolution: From Molecular Mechanisms to Therapeutic Perspectives

**DOI:** 10.3389/fphar.2020.00480

**Published:** 2020-04-08

**Authors:** Morena Zusso, Leanne Stokes, Stefano Moro, Pietro Giusti

**Affiliations:** ^1^Department of Pharmaceutical and Pharmacological Sciences, University of Padua, Padua, Italy; ^2^School of Pharmacy, University of East Anglia, Norwich, United Kingdom

**Keywords:** neuroinflammation, neurodegenerative diseases, microglia, cytokines, therapeutic targets

Neuroinflammation, the complex immune response of the central nervous system (CNS), when sustained, is a common denominator in the etiology and course of all major neurological diseases, including neurodevelopmental, neurodegenerative, and psychiatric disorders (e.g., Alzheimer's disease, AD; Parkinson's disease, PD; multiple sclerosis, MS; motor neuron disease; depression; autism spectrum disorder; and schizophrenia). Cellular (microglia and mast cells, two brain-resident immune cells, together with astrocytes) and molecular immune components (e.g., cytokines, complement and pattern-recognition receptors) act as key regulators of neuroinflammation (Skaper et al., 2012). In response to pathological triggers or neuronal damage, immune cells start an innate immune response with the aim to eliminate the initial cause of injury. However, when the cellular activity becomes dysregulated, it results in an inappropriate immune response that can be injurious and affect CNS functions. Thus, limiting neuroinflammation and microglia activity represents a potential strategy to alleviate neuroinflammation-related diseases.

The Research Topic collects 20 manuscripts, divided into five sections, that include both original research articles and reviews of the emerging literature and explore the role of neuroinflammation in various neurological diseases. There is particular attention dedicated to the relevant research exploring the mechanisms and mediators involved in the resolution of neuroinflammation. Our aim was to generate a valuable discussion contributing to identify new therapeutic targets in brain damage and providing new drug development opportunities for the prevention and treatment of CNS diseases involving neuroinflammation.

## Pathophysiology of Neuroinflammation

Guzman-Martinez et al. presented an overview of the various biochemical pathways involved in the neuroinflammatory cascade, focusing on the role of neuroinflammation in the process of protein alteration implicated in various neurodegenerative diseases, such as AD, PD, and Huntington disease. Herradon et al. highlighted the crosstalk between peripheral inflammation and neuroinflammation. The authors described cytokines such as pleiotrophin and midkine as key mediators in modulating both peripheral inflammation and neuroinflammation and they discussed the role of these cytokines in mediating both chronic neuroinflammatory conditions (i.e., neurodegenerative diseases) and peripheral inflammation. Furthermore, the existing therapeutics for overt neuroinflammation are reviewed and new perspectives suggested for the development of innovative strategies that focus on the modulation of the pleiotrophin-midkine pathway.

Oxidative stress is a critical and common feature of a wide spectrum of CNS pathologies and has been strongly implicated in microglial activation and neuroinflammation. Malko et al. provided an extensive review focusing on the role of the transient receptor potential melastatin-related 2 (TRPM2) channel, an oxidative stress-sensitive calcium-permeable cationic channel, in the activation of microglia and neuroinflammation. The authors emphasized that in CNS diseases microglia-related neuroinflammation is strongly attenuated in TRPM2-KO mice or in animals treated with a TRPM2 inhibitor. These results suggested that microglial TRPM2 represents a prospective novel therapeutic target for neuroinflammatory CNS diseases.

In the attempt to find new antiinflammatory treatments, Joffre et al. described the antineuroinflammatory and neuroprotective effects of n-3 long chain polyunsaturated fatty acids and the derived specialized proresolving mediators, with the aim to identify molecules derived from these fatty acids as possible protagonists of the antiinflammatory effects of polyunsaturated fatty acids.

## Neuroinflammation in AD and Memory Impairment

AD is the most common age-related neurodegenerative disease and is characterized by progressive memory decline and cognitive dysfunction. The impact of chronic inflammation is of relevance in the pathophysiology of AD, considering that subjects affected by inflammatory diseases have an increased risk of developing AD (Heneka et al., 2015). Dysregulated immunoactivity in AD has been widely studied and several papers point to microglia as legitimate drug targets in AD. In this context, Biber et al. provided a concise and informative review that reconciled some of the most recent updates in the understanding of microglia biology with drug discovery, noting that the dynamic properties of microglia and their location in the CNS make targeted small molecule therapies relatively difficult to develop. The authors proposed several microglia target candidates (e.g., CD33, kynurenine pathway, Toll-like receptors, the potassium channel K_Ca_3.1, etc.), with the optimistic hope that one of them could be used in developing drugs useful for AD therapy.

In the attempt to understand the mechanisms underlying the link between neuroinflammation and AD, several authors have studied the effects of some small molecules in animal models of AD. Bellozi et al. evaluated the effects of a 14 days oral treatment with dactolisib, a dual phosphatidylinositol 3 kinase (PI3K)/mammalian target of rapamycin (mTOR) inhibitor, in a transgenic mouse model of AD overexpressing the amyloid precursor protein (APP). Among the different pathways involved in the maintenance and progression of AD, an abnormal and continuous activation of PI3K/protein kinase B (Akt)/mTOR signaling contributes to disease progression, because of the disrupted clearance of amyloid β (Aβ) and tau, synaptic loss, and cognitive decline (O'neill, 2013; Heras-Sandoval et al., 2014). Dactolisib reduced social memory impairment, microglia activation in CA3 region of hippocampus, and the levels of hippocampal interleukin (IL)-10, suggesting that an adequate control of PI3K/Akt/mTOR pathway activation might have the potential to ameliorate AD signs and symptoms. Ano et al. demonstrated interesting effects of a short-term intake of iso-α-acids, which are bitter components in beer, in the reduction of the hippocampal inflammation and neural hyperactivation and memory impairment in a transgenic mouse model of AD.

Considering that chronic psychosocial stress represents a risk factor for AD, being associated with cognitive deficits, microglia priming, and inflammatory responses in adult brain (Piirainen et al, 2017), Wang Y. et al. focused on the relation between social stress and impairment of learning and memory in AD. The authors showed that icariin, a natural flavonoid extracted from *Epimedium brevicornum Maxim* (a traditional Chinese herb), attenuated restraint/isolation stress-induced memory damage, Aβ accumulation, and neuroinflammation by reducing microglia M1 activation and activating peroxisome proliferator-activated receptor γ in APP/PS1 mice.

Alagan et al. showed the neuroprotective and antiinflammatory effects of an ethanol extract of *Phyllanthus amarus* in an *in vivo* rat model of lipopolysaccharide (LPS)-induced memory impairment and neuroinflammation. Lykhmus et al. presented an interesting study on the effects of mesenchymal stem cells (MSCs) in a mouse model of memory impairment induced by LPS. Intravenously injection of MSCs or their conditioned media prevented α7 nAChR decrease, Aβ accumulation, and episodic memory decline induced by a single dose of systemic LPS in mice, suggesting that MSCs could be a potential therapeutic tool to treat neuroinflammatory-related cognitive pathology.

## Neuroinflammation in Psychiatric Disorders

Increasing evidence indicates that dysregulated or enhanced neuroinflammation is closely linked with the pathogenesis of psychiatric disorders, such as schizophrenia, anxiety and depression (Müller and Bechter, 2013; Weber et al., 2017). In a timely and comprehensive review, Müller considered the role of the intracellular adhesion molecule-1 (ICAM-1) in psychiatric illness. ICAM-1, expressed in microglia, astrocytes, and in endothelial cells of the CNS, is of particular interest from two interrelated reasons: it is a cell bound adhesion molecule, intimately associated with the blood brain barrier, and as a soluble protein it has been measured in blood or cerebrospinal fluid as a biomarker for several mental health disorders.

Depression is a recurrent, common, and potentially life-threatening psychiatric disease related to multiple causes. Hyperactivity of the hypothalamic-pituitary-adrenal axis, involved in the pathophysiology of depression, induces microglia activation and the overproduction of proinflammatory cytokines in the brain (Brites and Fernandes, 2015). Extensive research in recent years has been concentrated on the immuno-inflammatory mechanisms for the treatment of depression. Indeed, many antidepressants are endowed with antiinflammatory effects (Kenis and Maes, 2002). However, current antidepressants have important limitations, due to significant adverse reactions, poor compliance, and a high risk of relapse following drug withdrawal (Berwian et al., 2017). At present, natural plant products are offering attractive alternatives in antidepressant drug development. In this context, Ke et al. examined the effect of quercetin, a natural polyphenol with antiinflammatory and neuroprotective effects, in a rat model of LPS-induced depression. The authors showed that quercetin attenuated the inflammation induced depression-like behavior and improved learning and memory in LPS-challenged rats. These effects were associated with the reversal effects of quercetin on LPS-induced alteration of expression of brain derived neurotrophic factor, Copine 6, and TREM1/2 in the hippocampus and in the prefrontal cortex. Lee et al. found that myelophil, a 30% ethanol extract of Radix Astragali and Radix Salviae, reversed the depressive behavior in a mouse model of unpredictable chronic mild stress. Myelophil also decreased the over-activation of microglia and the inflammatory response in the hippocampus, and reversed the reduction of serotonergic function in the dorsal raphe nuclei and neurogenesis in the subgranular zone of hippocampus.

## Neuroinflammation in MS and Experimental Autoimmune Neuritis

MS is a CNS autoimmune and neurodegenerative disease that affects ~2.5 million people worldwide and causes a significant financial burden. Several experimental and clinical studies have revealed that microglia/macrophages actively participate in the course of the disease. In this context, Wang J. et al. provided a comprehensive review that attempted to discuss the role of macrophages and microglia in both healthy CNS and in MS. The authors discussed the possibility of a differential manipulation of CNS resident microglia and infiltrating macrophages to potentially target different mechanisms operating in MS and realize efficient treatments for the disease. Zhang F. et al. demonstrated that scopoletin, a phenolic coumarin found in some plants, improved the severity of the disease and prominently decreased inflammation and demyelination in an experimental autoimmune encephalomyelitis mouse model of MS, *via* nuclear factor-κB signaling.

Xu et al. examined the effect of oridonin, a diterpenoid compound extracted from *Rabdosia rubescens*, in an experimental autoimmune neuritis animal model. Oridonin ameliorated inflammatory disease progression and favored the disease outcome by inducing the switch of macrophages toward the antiinflammatory polarization state. Thus, oridonin could be considered a possible therapeutic candidate of inflammatory neuropathies.

## Neuroinflammation in Chronic Pain, Hyperammonia, Cardiac Arrest, and Intracerebral Hemorrhage

Chronic pain is caused by nerve damage which occurs during nerve compression, diabetes, inflammation, and shingles virus infection (Campbell and Meyer, 2006). Cytokines, chemokines, prostaglandins, and nitric oxide released from activated microglia and astrocytes in the dorsal horn of the spinal cord play important roles in the pathogenesis of chronic pain (Skaper et al., 2018). Therefore, several studies have targeted activated microglia to reduce pain hypersensitivity. In light of this, the manuscript by Song et al. showed the positive effect of GNF-2, a selective allosteric inhibitor of Bcr-Abl, initially developed as an anticancer drug, on neuroinflammation and associated pain pathogenesis, using different animal models of pain.

Malaguarnera et al. studied the effect of bicuculline, a GABA_A_ receptor antagonist, on hyperammonemic rats. In the hippocampus of these animals, GABAergic tone was increased contributing to some aspects of neuroinflammation. Glutamate transmission was altered and spatial learning and memory as well as anxiety were impaired. As expected, the treatment with bicuculline reduced hippocampal astrocytosis, but failed to control glial activation. Furthermore, the antagonist reduced the GABAergic tone and reversed the expression of the GluA1 and GluA2 subunits of AMPA receptors and of the NR2B subunit of NMDA receptors. The treatment with bicuculline also improved spatial learning, working memory and decreased anxiety in these rats.

The manuscript by Ma et al. explored the capacity of a bioactive annexin A1 short peptide to resolve neuroinflammation in a rat model of exsanguinating cardiac arrest treated by emergency preservation and resuscitation. The attenuation of cortical cell death and reduction of several biomarkers of neuroinflammation induced by the peptide were associated with the increased expression of sirtuin 3 and the upregulation of antioxidant pathways.

Zhang J. et al. indicated that a systemic treatment with simvastatin reduced polymorphonuclear neutrophil infiltration into brain tissue, ameliorated brain edema, and reduced proinflammatory mediator expression in perihematomal area in an animal model of neuroinflammation after intracerebral hemorrhage.

In conclusion, the Research Topic “Neuroinflammation and its resolution: from molecular mechanisms to therapeutic perspectives” presents the current state of knowledge about the involvement of microglia and neuroinflammation in neurological disorders and provides several new perspectives and therapeutic approaches for the development of novel pharmacological agents aimed at counteracting neuroinflammatory diseases.

Finally, it should be noted that in six manuscripts (~30%) of this Research Topic, the antiinflammatory effect of plant-derived products has been investigated. There is a high rate of failure in development of drugs for neurological diseases and for AD, in particular. New treatments are urgently needed, but even if progress is being made, we probably need to define new targets and developing new specific agents. In that regard, natural products could be a valuable source to provide candidates to be used in CNS diseases.

## Author Contributions

All the authors have contributed to the composition and the revision of this Editorial Article and approved it for publication.

## Conflict of Interest

The authors declare that the research was conducted in the absence of any commercial or financial relationships that could be construed as a potential conflict of interest.

## References

[B1] Berwian I. M. Walter H. Seifritz E. Huys Q. J. (2017). Predicting relapse after antidepressant withdrawal—a systematic review. Psychol. Med. 47, 426–437. 10.1017/S0033291716002580 27786144 PMC5244448

[B2] Brites D. Fernandes A. (2015). Neuroinflammation and depression: microglia activation, extracellular microvesicles and microRNA dysregulation. Front. Cell. Neurosci. 9, 476. 10.3389/fncel.2015.00476 26733805 PMC4681811

[B3] Campbell J. N. Meyer R. A. (2006). Mechanisms of neuropathic pain. Neuron 52, 77–92. 10.1016/j.neuron.2006.09.021 17015228 PMC1810425

[B4] Heneka M. T. Carson M. J. El Khoury J. Landreth G. E. Brosseron F. Feinstein D. L. (2015). Neuroinflammation in Alzheimer's disease. Lancet Neurol. 14, 388–405. 10.1016/S1474-4422(15)70016-5 25792098 PMC5909703

[B5] Heras-Sandoval D. Perez-Rojas J. M. Hernandez-Damian J. Pedraza-Chaverri J. (2014). The role of PI3K/AKT/mTOR pathway in the modulation of autophagy and the clearance of protein aggregates in neurodegeneration. Cell Signal. 26, 2694–2701. 10.1016/j.cellsig.2014.08.019 25173700

[B6] Kenis G. Maes M. (2002). Effects of antidepressants on the production of cytokines. Int. J. Neuropsychopharmacol. 5, 401–412. 10.1017/S1461145702003164 12466038

[B7] Müller N. Bechter K. (2013). The mild encephalitis concept for psychiatric disorders revisited in the light of current psychoneuroimmunological findings. Neurol. Psychiatry Brain Res. 19, 87–101. 10.1016/j.npbr.2013.04.004

[B8] O'neill C. (2013). PI3-kinase/Akt/mTOR signaling: impaired on/off switches in aging, cognitive decline and Alzheimer's disease. Exp. Gerontol. 48, 647–653. 10.1016/j.exger.2013.02.025 23470275

[B9] Piirainen S. Youssef A. Song C. Kalueff A. V. Landreth G. E. Malm T. (2017). Psychosocial stress on neuroinflammation and cognitive dysfunctions in Alzheimer's disease: the emerging role for microglia? Neurosci. Biobehav. Rev. 77, 148–164. 10.1016/j.neubiorev.2017.01.046 28185874

[B10] Skaper S. D. Giusti P. Facci L. (2012). Microglia and mast cells: two tracks on the road to neuroinflammation. FASEB J. 26, 3103–3117. 10.1096/fj.11-197194 22516295

[B11] Skaper S. D. Facci L. Zusso M. Giusti P. (2018). An Inflammation-Centric View of Neurological Disease: Beyond the Neuron. Front. Cell. Neurosci. 12, 72. 10.3389/fncel.2018.00072 29618972 PMC5871676

[B12] Weber M. D. Godbout J. P. Sheridan J. F. (2017). Repeated social defeat, neuroinflammation, and behavior: monocytes carry the signal. Neuropsychopharmacol. 42, 46–61. 10.1038/npp.2016.102 PMC5143478 27319971

